# Incidence and prevalence of patellofemoral pain: A systematic review and meta-analysis

**DOI:** 10.1371/journal.pone.0190892

**Published:** 2018-01-11

**Authors:** Benjamin E. Smith, James Selfe, Damian Thacker, Paul Hendrick, Marcus Bateman, Fiona Moffatt, Michael Skovdal Rathleff, Toby O. Smith, Pip Logan

**Affiliations:** 1 Derby Teaching Hospitals NHS Foundation Trust, London Road Community Hospital, Derby, United Kingdom; 2 Division of Rehabilitation and Ageing, School of Medicine, University of Nottingham, Nottingham, United Kingdom; 3 Department of Health Professions, Manchester Metropolitan University, Manchester, United Kingdom; 4 PhysioWorks (Sheffield), Sheffield Teaching Hospitals NHS Foundation Trust, Sheffield, United Kingdom; 5 Division of Physiotherapy and Rehabilitation Sciences, School of Health Sciences, University of Nottingham, Nottingham University Hospitals (City Campus), Nottingham, United Kingdom; 6 Research Unit for General Practice in Aalborg, Department of Clinical Medicine at Aalborg University, Aalborg, Denmark; 7 Department of Occupational Therapy and Physiotherapy, Department of Clinical Medicine, Aalborg University Hospital, Aalborg, Denmark; 8 Nuffield Department of Orthopaedics, Rheumatology and Musculoskeletal Sciences, University of Oxford, Oxford, United Kingdom; Queen Mary University of London, UNITED KINGDOM

## Abstract

**Background:**

Patellofemoral pain is considered one of the most common forms of knee pain, affecting adults, adolescents, and physically active populations. Inconsistencies in reported incidence and prevalence exist and in relation to the allocation of healthcare and research funding, there is a clear need to accurately understand the epidemiology of patellofemoral pain.

**Methods:**

An electronic database search was conducted, as well as grey literature databases, from inception to June 2017. Two authors independently selected studies, extracted data and appraised methodological quality. If heterogeneous, data were analysed descriptively. Where studies were homogeneous, data were pooled through a meta-analysis.

**Results:**

23 studies were included. Annual prevalence for patellofemoral pain in the general population was reported as 22.7%, and adolescents as 28.9%. Incidence rates in military recruits ranged from 9.7–571.4/1,000 person-years, amateur runners in the general population at 1080.5/1,000 person-years and adolescents amateur athletes 5.1%–14.9% over 1 season. One study reported point prevalence within military populations as 13.5%.

The pooled estimate for point prevalence in adolescents was 7.2% (95% Confidence Interval: 6.3%–8.3%), and in female only adolescent athletes was 22.7% (95% Confidence Interval 17.4%–28.0%).

**Conclusion:**

This review demonstrates high incidence and prevalence levels for patellofemoral pain. Within the context of this, and poor long term prognosis and high disability levels, PFP should be an urgent research priority.

**PROSPERO registration:**

CRD42016038870

## Introduction

There are over 100,000 primary care appointments a day in the United Kingdom (UK) for musculoskeletal (MSK) pain disorders [1], with an associated £7.4 billion cost annually to the UK economy through absenteeism [2]. In the United States an estimated 126.6 million Americans suffer from a musculoskeletal disorder, putting a burden onto the economy with an estimated cost of $213 billion annually through healthcare costs and sickness absence [3]. Knee pain is the second most prevalent condition, with patellofemoral pain (PFP) being considered one of the most common forms of knee pain [4], with a prevalence cited between 15% to 45% [5]. It is described as non-traumatic in nature, with diffuse anterior knee pain on activities that load the joint such as squatting, running, climbing and descending stairs [4].

Variations in reported incidence and prevalence may be due to differing populations assessed, inconsistencies in the diagnosis and lack of high quality evidence on which to base assessment [6,7]. PFP is thought to affect the general population [8], and more specifically adolescents [9], young active adults [10], elite athletes [11,12], and military recruits [13]; with higher incidence and prevalence rates often cited among females [13,14]. There is no definitive gold standard method to clinically diagnose PFP [15]. Diagnosis has historically been based on detailed subjective and objective assessments, with pain on a number of special tests including the patellofemoral compression test, palpation of the patella and pain of resisted knee extension [13,16–18]. It is likely that this method of diagnosis could under-estimate the true incidence or prevalence rates, since many people with PFP reduce or withdraw from their aggravating activity [19], and consequently pain on palpation may only identify those with higher levels of pain, or still participating in activities. Alternatively, due to high sensitivity of historical tests, it may be that this approach results in an over-estimation instead [20]. Recently PFP has been re-defined as: self-reported pain around or behind the patella aggravated by activities that loads the joint, e.g. squats, stairs, running or jumping; with special tests and pain on prolonged rest additional but not essential [4].

To date, no systematic reviews have been published on the incidence and prevalence for PFP; with publications often employing an indirect course of secondary and even tertiary referencing when citing incidence or prevalence data for PFP [7]. In relation to clinical decision-making, and the allocation of healthcare and research funding, there is a clear need to accurately understand the epidemiology of this problem. Therefore, in the context of the current uncertainty regarding PFP, this systematic review synthesises epidemiological data using a contemporary case definition and clear population classifications [4], to gain an understanding of incidence and prevalence data for this condition.

## Method

This systematic review followed the recommendations of the meta-analyses in observational studies (MOOSE) guidance statement [21], the recommendations of the PRISMA statement where relevant [22], and was registered with the International Prospective Register of Systematic Reviews (PROSPERO reference CRD42016038870).

### Data sources and search strategy

An electronic database search was conducted on titles and abstracts from inception to June 2017 using the following databases: Medline via PubMed, EMBASE, the Cumulative Index to Nursing and Allied Health Literature (CINAHL), and Web of Science. For the keywords search strategy used see Table 1. The database searches were accompanied by hand searches of the reference list of included articles, as well as contacting authors for all included and potentially included studies. The grey literature and ongoing studies were searched using the following databases: OpenGrey, WHO International Clinical Trials Registry Platform, ClinicalTrials.gov and the NIHR portfolio.

**Table 1:** Search strategy.

	Search Term
**1**	”anterior knee pain” or “AKP” or “patellofemoral pain syndrome” or “PFPS” or “patellofemoral pain” or “PFP”
**2**	Inciden$ or prevalen$ or cohort$ or prospective or epidemiolog$ or trial
**3**	1 and 2 limited to English language

Inclusion criteria included study population of any age and any setting with signs and symptoms of PFP, defined as; anterior or retropatellar pain reported on at least two of the following activities; prolonged sitting, ascending or descending stairs, squatting, jumping and running [4]. There was no restriction on the type of setting for potential included papers.

Exclusion criteria included: if the study population was selected from a specific disease area (e.g. diabetes, rheumatoid arthritis, osteoarthritis); if the study population comprised of participants with other knee pathology (e.g. knee ligamentous instability, history of patella dislocations, true knee locking or giving way, patella or iliotibial tract tendinopathy, osteoarthritis).

Included studies were required to report incidence or prevalence data, and had to be published in English or where an English translation was available.

### Study selection

One reviewer (BES) undertook the searches. Titles and abstracts were screened by one reviewer (BES), with potential eligible papers retrieved and independently screened by two reviewers (BES & JS). Initial inclusion agreement was 83%. Five disagreements were due to case definition [9,12,23–25], and were discussed and resolved through consensus. Seven further case definition disagreements not resolved through consensus were resolved through a third reviewer (PH) [26–32].

### Data extraction

One reviewer (BES) extracted data relating to study design, population and setting, case definition, incidence and prevalence data, which was independently verified by a second reviewer (DT).

### Quality appraisal

In the absence of any validated quality assessment tools [33], two reviewers (BES & JS) independently appraised methodological quality using a tool developed by Hayden et al. [34] for the evaluation of the quality of prognostic studies in systematic reviews, and adapted by Luime et al. [35] for evaluation of the quality of epidemiological studies in systematic reviews. This assessed appropriateness and reporting of: the study population, case definition, and the response rate and follow-up of the cohort. To be judged as ‘high quality’, all three criteria had to be met; with male and females represented, a clear reproducible case definition relevant to our inclusion criteria and a response rate above 75%. Percentage agreement between the two reviewers was 94%, all disagreements were discussed and resolved through consensus.

### Data synthesis

Study heterogeneity was assessed through visual examination of the data extraction table on details related to participant characteristics, case definition, study design and process of the included studies. If heterogeneous, data were analysed narratively to assess trends in prevalence and incidence across the studies. When data allowed, incidence rates were converted to cases per 1,000 person-years, with associated 95% confidence intervals (CI) [36]. Where studies were homogeneous, data were pooled through a meta-analysis. Statistical heterogeneity was assessed using the ***I***^2^ statistic where 0% to 25% was low, 26% to 74% moderate and 75% and over high statistical heterogeneity [37]. When outcomes presented with low statistical heterogeneity, data were pooled using a fixed-effects model, with moderate or high statistical heterogeneity a random-effects model was adopted. All data analyses were performed using Stata version 14.0 (College Station, TX, USA) [38].

## Results

### Study selection

**Fig 1:**
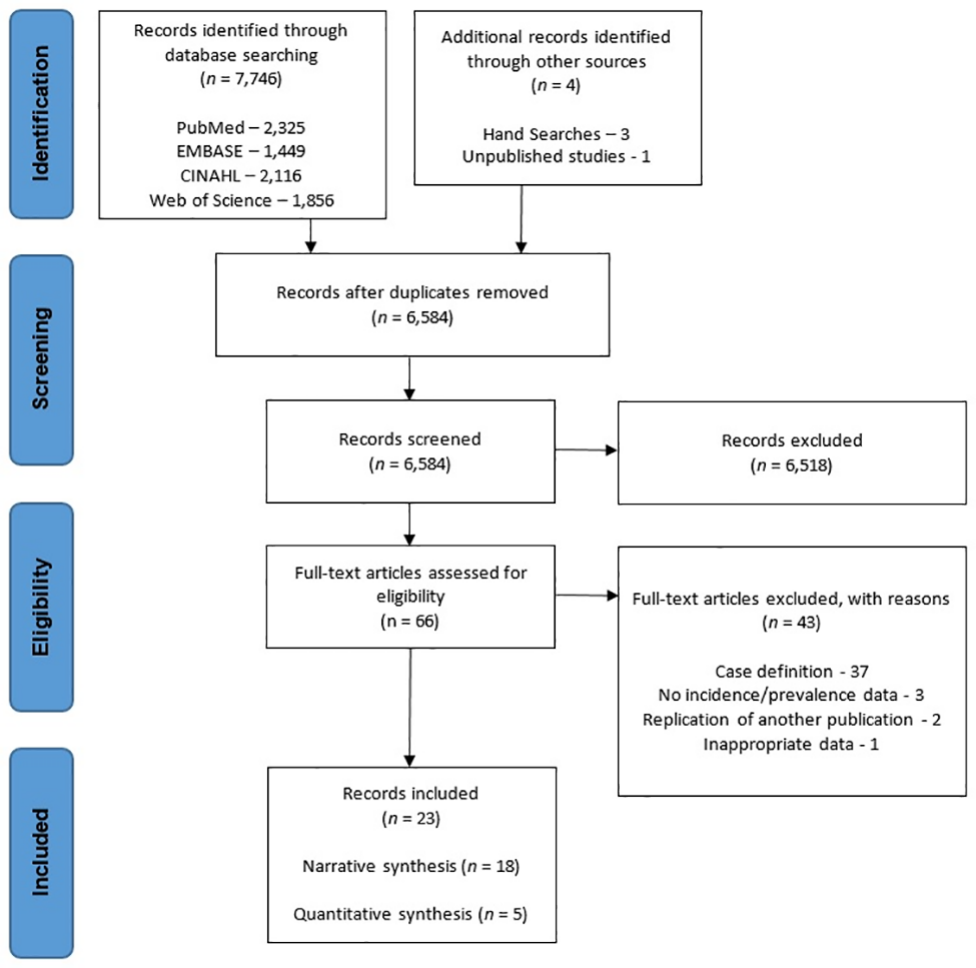
PRISMA 2009 flow diagram.

The search results are presented in Fig 1. From a total of 7,746 titles 66 papers were potentially eligible. One unpublished trial was identified, however the author declined to share the details. 43 full-text articles were excluded; 37 due to case definition not meeting criteria [10,25,39–73], three due to no prevalence or incidence data being recorded [23,74,75], and two because they were a replication of another included study [76,77]. In one study participants were tested longitudinally over multiple years, with participants being eligible to enrol multiple times, and therefore was excluded [78]. 23 studies met the eligibility criteria and were included in the final review, 12 reporting incidence data [13,16–18,26,28,30,32,79–82], and 13 reporting prevalence data [5,9,11–13,16,24,27,29,31,83–85]. Of the included 23 papers, 12 authors were contacted for clarification on: raw data extraction [11,26,28,29,79,80,84], and participant information [5,12,13,18,31]. Eight responded and gave further details, where available [5,12,13,28,29,31,80,84]. The authors that were uncontactable, or did not have available information account for the ‘unknown’ items in the characteristics table.

### Study characteristics

A summary of the main characteristics of the included studies, with the main results can be found in Tables 2 and 3 for incidence and prevalence respectively. Six papers within the military setting (*n* = 4,199) reported incidence data [13,17,32,79–81], two papers reported incidence data within the general adult population setting (*n* = 140) [26,82], and four papers reported incidence data within the general adolescent population (*n* = 985) [16,18,28,30]. One paper within the military setting (*n* = 1,525) reported prevalence data [13], three papers reported prevalence data within the general adult population setting (*n* = 1,1011) [5,24,83], six papers reported prevalence data the general adolescent population setting (*n* = 5,090) [9,16,27,29,31,84], and three papers reported prevalence data the elite athlete setting (*n* = 568) [11,12,85].

**Table 2:** Incidence.

Study	Quality score	Study population	Sample Size	Case definition	Response rate	Results
**Military**						
Boling (2010) [13]	3/3	United States Naval Academy (USNA) (39.8% female; mean age unknown, range 18–25*).	1,525	Retropatellar pain of any duration with two of the following activities: ascending/descending stairs, hopping/jogging, prolonged sitting with flexed knees, kneeling, and squatting. Plus one of the following: pain on palpation of medial or lateral patellar facets, or pain on palpation of the anterior portion of the medial or lateral femoral condyles.	1,319/1,525 (86.5%)	22/1,000 person-years (95% CI: 15/1,000, 29/1,000 person-years). Female incidence was 33/1,000 person-years (95% CI: 20/1,000, 45/1,000 person-years) and in males was 15/1,000 person-years (95% CI: 7/1,000, 22/1,000 person-years).
Coppack (2011) [79]	2/3	British Army recruits, United Kingdom (27.9% female, mean age 19.6).	743	Pain from at least 2 of the following: prolonged sitting, stair climbing, squatting, running, kneeling, and hopping/jumping; insidious onset of symptoms unrelated to a traumatic incident; and presence of pain on palpation of the patellar facets, on step down from a 25-cm step, or during a double-legged squat.	743/743 (100%)	14 week incidence 36 / 743 (4.8%; 95% CI, 3.5–6.7). 180/1,000 person-years (95% CI: 127.9/1,000, 246.5/1,000 person-years).
Kaufman (1999) [80]	2/3	United States Navy Recruits. (100% male; mean age 22.5 years)	449	Ill-defined ache of insidious onset localized to the peripatellar area, plus pain on palpation of patella and peripatellar soft tissues.	449/449* (100%)	25 week incidence 35/449 (7.8%). 162.1/1,000 person-years (95% CI: 114.7/1,000, 223.0/1,000 person-years).
Milgrom (1991) [17]	1/3	Infantry recruits, Israel (100% male; age unknown).	390	Subjective complaint of anterior knee pain, non-traumatic, with objective finding of pain on patellofemoral compression test and palpation of patella borders.	390/390 (100%)	14 week incidence 60/390 (15.4%). 571.4/1,000 person-years (95% CI: 439.9/1,000, 730.5/1,000 person-years).
Thijs (2007) [32]	3/3	Belgian Royal Military Academy recruits (22.6% female; mean age 19).	84	Two of the following: pain on direct compression of the patella with the knee in full extension, tenderness of the posterior surface of the patella on palpation, pain on resisted knee extension, or pain with isometric quadriceps muscle contraction.	84/105 (80%)	6 week incidence 36/84 (42.9%). 9.7/1,000 person-years (95% CI: 6.9/1,000, 13.3/1000 person-years).
Wills (2004) [81]	3/3	British Army Recruits (95.2% male; median age 19.4)	1,008	Pain around the anterior aspect of the knee, insidious onset and no evidence of trauma	926/1,008 (91.9%)	12 week incidence 81/926 (8.7%). 379.1/1,000 person-years (95% CI: 303.0/1,000, 468.7/1,000 person-years).
**General Adult Population**						
Devan (2004) [26]	2/3	Female amateur collegiate hockey, basketball and athletic athletes, USA (mean age 19.4).	63	Pain in or under patella while running, going up or down stairs; with diffuse pain on palpation.	53/63 (84.1%)	1 athletic season incidence 1/53 (1.9%).
Thijs (2011) [82]	2/3	Female novice recreational runners on a 10 week start to run programme, Belgium (mean age 38.4)	77	Retropatellar pain during and/or after activities such as running, squatting, kneeling, going up and down stairs, cycling, prolonged sitting with the knee in flexion, or rising from a seated position. And 2 of the following: pain while compressing the patella, tenderness of patella on palpation, painful resisted knee extension and pain when isometrically contracting the quadriceps 15° flexion.	77/77 (100%)	10 week incidence 16/77 (20.8%). 1080.5/1,000 person-years (95% CI: 639.6/1,000, 1717.0/1,000 person-years).
**General Adolescents Population**						
Finnoff (2011) [28]	2/3	High School runners aged 14–18, USA (45.9% female; mean age 16)	98	Anterior knee pain that was exacerbated by deep knee bending and/or climbing stairs plus pain on one of the following: (1) pressure over the subject’s distal quadriceps tendon combined with active contraction of his or her quadriceps muscle (patellar grind test) or (2) direct palpation of the medial or lateral patellar facets.	98/1500 (6.5%)	1 running season incidence 5/98 (5.1%).
Herbst (2015) [30]	1/3	Female adolescent basketball players in middle and high school, USA (mean age 12.7 years).	255	Anterior Knee Pain Scale score < 100; International Knee Documentation Committee (IKDC) form, standardized history and physician-administered physical examination.	255/329 (77.5%)	1 season incidence 38/255 (14.9%). 0.97 per 1,000 athletic exposures (1 game or training session).
Myer (2010) [16]	2/3	Female adolescent athletes in middle and high school, USA (mean age 13.4 years)	152	Anterior Knee Pain Scale score < 100; knee pain with or shortly following activity and also if anterior knee tenderness was recent.	145/152 (95.4%)	1 season incidence 14/145 (9.7%). 1.09 per 1,000 athletic exposures (1 game or training session).
Witvrouw (2000) [18]	1/3	Students taking physical education, aged 17–21 in Belgium (sex unknown; mean age 18.6)	480	Retropatellar pain > 6 weeks during physical activities such as jumping, running, squatting, and going up or down stairs. Plus two of the following; pain on direct compression of the patella, tenderness of the posterior surface of the patella, pain on resisted knee extension, and pain with isometric quadriceps contraction.	282/480 (58.8%)	2 year incidence 24/282 (8.5%). 42.6/1,000 person-years (95% CI: 27.9/1,000, 62.4/1,000 person-years). Female incidence was 13/131 (9.9%), 49.6/1,000 person-years (95% CI: 27.6/1,000, 82.7/1,000 person-years); male was 11/151 (7.3%), 36.4/1,000 person-years (96% CI: 19.2/1,000, 63.3/1,000 person-years).

*Information not within publication, authors contacted for clarification.

**Table 3:** Prevalence.

Study	Quality score	Study population	Sample Size	Case definition	Response rate	Results
**Military**						
Boling (2010) [13]	3/3	United States Naval Academy (USNA) (39.8% female; mean age unknown, range 18–25*).	1,525	Retropatellar pain of any duration with two of the following activities: ascending/descending stairs, hopping/jogging, prolonged sitting with flexed knees, kneeling, and squatting. Plus one of the following: pain on palpation of medial or lateral patellar facets, or pain on palpation of the anterior portion of the medial or lateral femoral condyles.	1,525/1,525 (100%)	Point prevalence of PFPS was 13.5% (95% confidence interval (CI): 11.7%, 15.3%]. For females and males it was 15.3% (95% CI: 13.7%, 16.9%) and 12.3% (95% CI: 11.1%, 13.4%), respectively.
**General Adult Population**						
Dey (2016) [83]	3/3	Community within the UK. Convenience sample of attendance at a University science fair (53% female; mean age 30).	111	Anterior knee or retropatellar pain, often bilateral, of insidious onset present for at least a month and associated with pain or difficulty with prolonged sitting or activities which load the patellofemoral joint, e.g., ascending or descending stairs, running and squatting. Positive diagnosis identified through a self-report questionnaire (SNAPPS- Survey instrument for Natural history, Aetiology and Prevalence of Patellofemoral pain Studies)	110/111 (99%)	Annual prevalence 25/110 (22.7%). Females 67%; males 33%.
Roush (2012) [5]	3/3	18–35 year old females, general population*, USA (mean age 24.7)	769	Anterior Knee Pain Scale score < 83	724/769 (94.1%)	Point prevalence was 12–13%*.
Weiss (1985) [24]	3/3	Amateur multi-day cyclist in USA (69% male; mean age 41.4).	132	Self-reported complaint of patella pain during a cycling event. Tenderness of posterior aspect of patella during flexion and extension.	113/132 (86%)	Point prevalence was 35%.
**General Adolescents Population**						
Fairbank (1984) [27]	1/3	13–17 year-old students, randomly selected from a comprehensive school in the United Kingdom (49% female, mean age 14.7)	446	11 point questionnaire, including: Do you like playing sport? Have you had painful knees in the last year? Do your knees hurt climbing stairs? Do your knees hurt coming downstairs? Where do you feel the pain in your knees? Does your knee hurt after sitting for a long time? Does your knee hurt only after a lot of exercise?	446/1850 (24.1%)	Annual prevalence 129/446 (28.9%).
Hall (2015) [29]	2/3	Female adolescent athletes in middle and high school, USA (mean age 14.0).	546*	Assessment included the Anterior Knee Pain Scale (AKPS), International Knee Documentation Committee (IKDC) form, standardized history and physician-administered physical examination.	546/546*	Point prevalence 151/546* (28%).
Molgaard (2011) [9]	3/3	16–18 year-old students at one local high school in Denmark (mixed sex; mean age 16.9)	299	Anterior knee pain during physical activity for at least 1 month and pain in at least two of the following four tests: isometric contraction of quads, concentric extension against resistance, palpation of joint line, and compression of the patella.	227/299 (76%)	Point prevalence 13/227 (5.7%). Females 69%; males 31%.
Myer (2010) [16]	2/3	Female adolescent athletes in middle and high school, USA (mean age 13.4 years).	240	Anterior Knee Pain Scale score < 100; knee pain with or shortly following activity and also if anterior knee tenderness was recent.	240/240 (100%)	Point prevalence was 39/240 (16.3%).
Rathleff (2014) [84]	3/3	Population-based cohort of students from secondary schools, Denmark, aged 15–19 years (64.9% female; mean age 17.2).	2,200	Insidious onset of anterior knee or retropatellar pain for at least the past 6 weeks; pain provoked by at least 2 of the following activities: prolonged sitting or kneeling, squatting, running, hopping, or stair walking and tenderness on palpation of the patella.	2,220/2846* (77.3%)	Point prevalence 153/2,062 (7.4%)*.
Steinberg (2012) [31]	1/3	Non-professional female dancers, aged 8–20, Israel (mean age 13.7 years*).	1,359	Pain reproduced during clinical examination; knee swelling was evident, or a positive grinding sign and/or a positive Patellar Inhibition Test (PIT) was obtained when the knee and especially the patella were palpated, contracted and stretched.	1,359/1,359 (100%)	Point prevalence 321/1,359 (23.6%).
**Elite Athletes**						
Clarsen (2010) [12]	2/3	Professional cycling; 7 training camps (100% male*; mean age 26)	109	Cyclist reported complaint of anterior knee pain in the last 12 months, of any duration. Cyclist reported complaint of anterior knee pain in the last 12 months, >30 days	109/109 (100%)	Annual prevalence 39/109 (35.8%). Annual prevalence 7/109 (6.4%).
Nejati (2010) [11]	1/3	Female athletes participating in 3^rd^ Iranian Sports Olympiad (mean age 21.6, range 15–35).	418	Non traumatic anterior knee pain of at least 3 months duration that was felt retropatellar or peripatellar and was aggravated by descending or ascending stairs, squatting or prolonged sitting.	418/unknown	Point prevalence was 70/418 (16.7%).
Winslow (1995) [85]	1/3	University female ballet dancers, USA (mean age unknown)	41	Pain in front of or under the knee cap with 3 out of 5: associated with kneeling; squatting; during stair climbing; sensations of cracking/grinding or with incidents of joint locking or "catching."	41/unknown	Point prevalence was 12/41 (29.3%).

*Information not within publication, authors contacted for clarification.

As a result of study heterogeneity, with the exception of five studies that reported prevalence data in the adolescent population, a narrative synthesis was conducted.

### Quality appraisal

The results of the methodological quality appraisal can be found in Table 4. 43.5% (10/23) of the included studies were high quality (quality score = 3/3), according to our definition. 26.1% (6/23) recorded a quality score of 2/3, and seven studies (30.4%) recorded a score of 1/3. The main risk of bias and low methodological quality was due to ten studies having populations comprising only male or female participants, and one study not describing the participant’s sex [11,16–18,26,29–31,80,82,85]. Three studies had a response rate of below 75% [18,27,28]; two had an unknown response rate [11,85]; and four studies had imprecise case definitions [17,27,30,31]; all were scored low accordingly.

**Table 4:** Quality appraisal.

	The study sample represents the population of interest on key characteristics	Was there an adequate response rate?	Was the case definition specified and is it reproducible?
Boling (2010) [13]	✓	✓	✓
Clarsen (2010) [12]	✓	✓	✓
Coppack (2011) [79]	✓	✓	✓
Devan (2004) [26]	X	✓	✓
Dey (2016) [83]	✓	✓	✓
Fairbank (1984) [27]	✓	X	X
Finnoff (2011) [28]	✓	X	✓
Hall (2015) [29]	X	✓	✓
Herbst (2015) [30]	X	✓	X
Kaufman (1999) [80]	X	✓	✓
Milgrom (1991) [17]	X	✓	X
Molgaard (2011) [9]	✓	✓	✓
Myer (2010) [16]	X	✓	✓
Nejati (2010) [11]	X	Unknown	✓
Rathleff (2014) [84]	✓	✓	✓
Roush (2012) [5]	✓	✓	✓
Steinberg (2012) [31]	X	✓	X
Thijs (2011) [82]	X	✓	✓
Thijs (2007) [32]	✓	✓	✓
Weiss (1985) [24]	✓	✓	✓
Wills (2004) [81]	✓	✓	✓
Winslow (1995) [85]	X	Unknown	✓
Witvrouw (2000) [18]	Unknown	X	✓

✓, yes; X, no

### Military

#### Incidence

Five studies reported incidence rates for military recruits, with a predominantly male population, that ranged from 9.7–571.4 cases per 1,000 person-years [17,32,79–81]. One study, with a mixed female and male military population, reported an incidence rate of 22 cases per 1,000 person-years, with female recruits being reported as 33 and males as 15, cases per 1,000 person-years [13].

#### Prevalence

One study with a mixed female and male military recruit population reported a point prevalence of 13.5%, females 15.3% and males 12.3% [13].

### General adult population

#### Incidence

One study with novice recreational female runners recorded a 10-week incidence rate of 1080.5 cases per 1,000 person-years [82]. One study with female amateur collegiate athletes (mean age 19.4) reported an athletic season incidence rate of 1.9%.

#### Prevalence

Annual prevalence in the general population was reported as 22.7%, with the annual prevalence in females 29.2% and males 15.5% [83]. Point prevalence in females was reported as 12% to 13% [5]. Point prevalence during a multi-day amateur cycling event for mixed male and female was reported as 35% [24].

### General adolescents population

#### Incidence

Two studies recorded the incidence rate over one season for female adolescent athletes as 9.7%–14.9%, or 0.97–1.09 per 1,000 athletic exposures [16,30], and one study recorded the incidence rate over two seasons with adolescents participating in physical education (sex unknown) as 42.6 cases per 1,000 person-years [18]. One mixed sex study of high school runners reported the incidence rate over one running season as 5.1% [28].

#### Prevalence

Two studies reporting point prevalence (Molgaard [9] and Rathleff [84]) on mixed male and female adolescents were deemed suitably homogenous, and accordingly were pooled in a meta-analysis. Statistical heterogeneity was negligible (*I*^2^ = 5.4%), and the pooled estimate of point prevalence using a fixed effects model was 7.2% (95% CI 6.2%–8.3%). Point prevalence in female only adolescents was reported as 16.3% [16].

Three studies reporting point prevalence (Hall [29], Myer [16], and Steinberg [31]) on female only adolescent athletes were deemed suitably homogenous, and accordingly were also pooled in a meta-analysis. Statistical heterogeneity was high (*I*^2^ = 85.7%), and the pooled estimate of point prevalence using a random effects model was 22.7% (95% CI 17.4%–28.0%).

One study if mixed sex adolescent reported an annual prevalence of 28.9% [27].

### Elite athletes

#### Prevalence

One study with professional male cyclists reported an annual prevalence of 35.7% with symptoms of any duration, and 6.4% with symptoms lasting greater than 30 days [12]. One study of female athletes (mean age 21.6) at the 3rd Iranian Sports Olympiad reported point prevalence of symptoms greater than 3 months of 16.7% [11]; and another with female university ballet dancers reported point prevalence (of unknown duration) as 29.3% [85].

## Discussion

### Summary of main findings

The results of this systematic review confirm that PFP is a common pathology among adolescents, the general population, and those with high levels of activity, such as elite athletes and military populations. Point prevalence within military populations is reported as 13.5% [13]; female general populations 12% to 13% [5]; multi-day amateur cyclists 35% [24]; and female elite sports 16.7% to 29.3% [11,85]. It was calculated through meta-analysis to be 7.2% in mixed sex adolescents, and 22.7% in female amateur athletes. Annual prevalence in the general population is reported as 22.7% [83]; in professional cyclists it is reported as 35.7% [13]; and in general adolescent population it is reported as 28.9% [27]. No studies that were included within our review reported life-time prevalence.

To our knowledge, this is the first review to systematically evaluate and synthesise incidence and prevalence data for PFP. Comparison between studies was fulfilled in relation to age, sex, and activity levels (general population, military and elite athletes).

### Clinical implications

Patellofemoral pain is often cited as an overuse injury [86], with short periods of overuse or an increase in physical activity thought be a particular risk factor [87]. Within the military population there was low agreement on incidence rate, with predominantly male recruits reported in five studies at 9.7–571.4 cases per 1,000 person-years [17,32,79–81]. Of note is the study with the highest reported incidence (571.4/1,000 person-years) originated from a country with military conscription [17], and may have a population comprising of participants not accustomed to intense periods of physical activity. Studies with lower reported incidences (9.7–349.1/1,000 person-years) were from countries without conscription, where high levels of physical fitness are a requirement of recruitment [13,32,79–81], and so may contain participants more accustomed to intense periods of physical activity. Within the general population the 10-week incidence for novice runners was comparable to the incidence in conscripted military recruits, at 1080.5 cases per 1,000 person-years [82]. These data seem to be in agreement with the model that attributes short periods of unaccustomed high levels of physical activity as a risk factor for development of PFP. Contemporary thinking in relation to training loads and injury risk challenges the idea that PFP is simply an overuse injury, with evidence suggesting that under-training may be a risk factor for an increase in injury risk in athletes [88]. Exposure to appropriate training loads and periodisation, without ‘spikes’ in training, is thought to be one method of risk management [88].

There was some consistency in the data relating to ratios of females to males, seemingly confirming the commonly cited claim that females are twice as likely to develop PFP than males [14]. One study demonstrated that females were approximately twice as likely to develop PFP as males during military training, however the same study also demonstrated no statistical difference in point prevalence between males (12.3%) and females (15.3%) (*p* = 0.09) prior to the start of the training programme; suggesting that the transition to elite military fitness from the general population is an important factor in PFP [13]. Another study reported point prevalence within the general population as 29.2% in females and 15.5% males [83]. Prevalence comparison between sexes for adolescents also demonstrates this phenomenon, with one study showing that females made up 69% of participants with PFP, compared with 31% in males [9]. This is confirmed by our pooled estimates of point prevalence, with mixed sex being calculated at 16.3% and female only at 22.7%.

These data may be used to identify possible populations who are at risk, which may help in relation to clinical decision-making, and the allocation of healthcare and research funding, such as adolescents and people attempting to increase physical activity levels. These populations may be at increased risk of developing of PFP, with subsequent development of pain and physical disability, and possible withdrawal from physical activity. As a consequence having reduced physical activity levels with loss of associated health benefits [89–91].

### Research implications

There appears to be a large discrepancy with research funding and priorities for PFP compared with other knee conditions. For example, there have been over 14,000 papers indexed in MEDLINE for knee osteoarthritis in the last 20 years, with only 1,500 papers indexed on PFP. Yet despite these large differences, incidences rates for PFP far exceed those reported for knee osteoarthritis. This review found reported incidences rates across all populations of 9.7–1080.5 cases per 1,000 person-years; based on primary-care data in Spain the incidence rates for knee osteoarthritis is reported as 8.3 cases per 1,000 person-years for females and 4.6 cases per 1,000 person-years for males, far lower than that of PFP [92]. Furthermore, disability, function and pain scores are comparable; disability and function as measured with the Knee injury and Osteoarthritis Outcome Scores (KOOS) [93], are similar with both conditions [84,94,95]; likewise pain on activity, as measured on a 100mm visual analogue scale, is equivalent [96,97]. Additionally, PFP often affects younger populations, with a significant degree of persistence, potentially making it a much more significant problem, with work absenteeism, and long term health implications through loss of physical activity [89–91].

One of the barriers research and healthcare funding faces for PFP is that historically it has been labelled a “benign, self-limiting condition” [98]. An influential 1985 cohort study by Sandow and Goodfellow [98], that followed 54 adolescents for two to eight years with a new diagnosis of PFP, concluded that a policy of non-intervention was justified in the management of this condition, and that the condition improved over time with few reporting disability. This interpretation contrasts with the APA2011 cohort from Denmark [99]. They demonstrated that at two year follow-up, adolescents with PFP are more likely to still be reporting pain than people with other knee conditions [99]. Indeed, a recent re-analysis of Sandow and Goodfellow’s data does not seem to support their own conclusions, with Luhann et al. [100] highlighting that of the original 54 adolescents, 94% still had pain at final follow-up, with 54% reporting same or worse severity of symptoms. This pattern of poor long term prognosis continues in the adult populations, with a large proportion (> 50%) of people still reporting pain and dysfunction five to eight years after a six weeks evidenced based treatment programme [101]; yet the impression that PFP is a benign and self-limiting condition, with non-intervention advised, has continued to guide funders and stakeholders decision making for decades [102]. In the context of the high incidence and prevalence numbers, poor long term prognosis and high disability levels, PFP should be an urgent research priority.

There is a pressing need for new studies into this condition. For example, advocates of qualitative research methods suggest that qualitative inquiry can disclose the lived experience of people with pain; and therefore be used to understand patient motivation, social engagement and provide a wealth of information about the sociocultural context to pain [103,104]; to date no qualitative body of work has been published on PFP. Qualitative inquiry can provide an insight that may lead to development of ideas and hypothesis generation within the context of the contemporary biopsychosocial model of pain. This could then be used to develop new conservative management approaches that could then be tested with efficacy and effectiveness trials.

### Strengths and limitations of included trials

A systematic and rigorous approach was taken to identify relevant studies, which included electronic database searching, hand searching, citation searching; with endeavour to find un-published studies.

The main sources of heterogeneity within the included studies were likely to be from difference in populations and ages. Other potential sources of heterogeneity are different study design methodologies, for example the nature of measures such as point or period, and differences in case definitions. There was no consistency within the included studies on the case definition used, with no two the same. Historically PFP was considered a separate pathology to intra-articular pathologies such as: bursitis, plica syndromes and chondromalacia patellae [105]; however several studies recently have demonstrated that structural abnormalities of the patellofemoral joint on Magnetic Resonance Imaging (MRI) are not associated with PFP [106,107], and a recent consensus statement from the 4th International Patellofemoral Pain Research Retreat defined PFP subjectively only, with no essential objective findings [4]. However, only seven of the included 23 studies in this review had a contemporary case definition that omitted objective findings [11,16,27,81,83–85].

### Limitations of this review

The study presented with two key limitations. For pragmatic reasons only one reviewer screened titles and abstracts. An extensive literature search was carried out, with two reviewers independently screening full-texts for inclusion, and two reviewers independently extracting the data. An attempt was made to retrieve unpublished trials; however it may be that not all trials were retrieved, particularly considering we did not search for papers published in languages other than English. It is likely that the inclusion of such data could influence the estimates of incidence and prevalence for PFP.

## Conclusion

PFP is a common condition, with approximately one in 10 military recruits and one in 14 adolescents suffering at any one time; and one in five of the general population experiencing pain within the last year. Due to a paucity of evidence uncertainty remains with regards to these estimates of incidence and prevalence, and further published or unpublished work is likely to revise our estimates. There is some consistency with data showing females are twice as likely to experience PFP as males. In the context of high incidence and prevalence numbers, poor long term prognosis and high disability levels, PFP should be an urgent research priority. GPs need to be aware of high risk groups, such as adolescents and adults increasing physical activity levels, and the persistent nature of the problem, and ensure timely referrals to physiotherapy to maintain physical activity levels.

## Supporting information

S1 File PRISMA checklist. (DOCX)

S1 Fig PRISMA flowchart. (TIFF)
