# Supplementary figures and images for: Incidence and prevalence of patellofemoral pain: A systematic review and meta-analysis
Source: PLoS One. 2018 Jan 11;13(1):e0190892. doi: 10.1371/journal.pone.0190892 (PMC5764329; doi:10.1371/journal.pone.0190892)

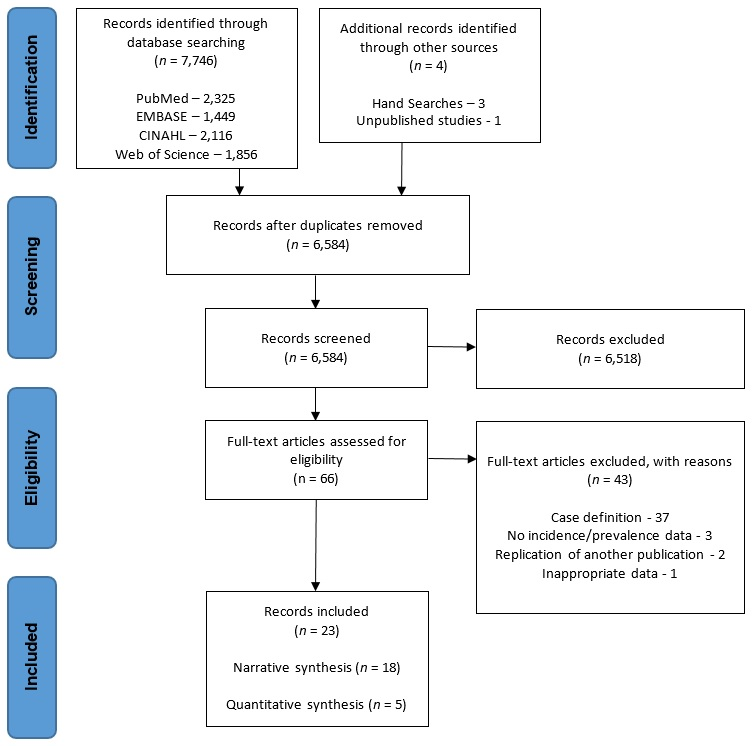

Supplement: S1 Fig — (TIFF) [file pone.0190892.s002.tiff]
